# Integrating health literacy into a theory-based drug-use prevention program: a quasi-experimental study among junior high students in Taiwan

**DOI:** 10.1186/s12889-021-11830-5

**Published:** 2021-09-28

**Authors:** Li-Chen Lin, Chiu-Mieh Huang, Hsiao-Pei Hsu, Jung-Yu Liao, Cheng-Yu Lin, Jong-Long Guo

**Affiliations:** 1 Department of Health Promotion and Health Education, College of Education, National Taiwan Normal University, No. 162, Section 1, Heping E. Road, 106308 Taipei, Taiwan; 2 Department of Nursing, Kang-Ning General Hospital, No. 26, Lane 420, Section 5, Chenggong Road, Neihu District, 114050 Taipei, Taiwan; 3 Institute of Clinical Nursing, College of Nursing, National Yang Ming Chiao Tung University, No. 155, Section 2, Li-Nong Street, 112304 Taipei, Taiwan; 4 Department of Public Health, Kaohsiung Medical University, No. 100, Shiquan 1st Road, Sanmin District, 807378 Kaohsiung, Taiwan; 5 Department of Radio, Television & Film, Shih Hsin University, No. 1, Lane 17, Section 1, Muzha Road, Wenshan District, 116002 Taipei, Taiwan

**Keywords:** Adolescents, School-based intervention, Health literacy, Drug-use prevention; theory of planned behavior

## Abstract

**Background:**

In Taiwan, illegal drug use is a critical health problem during adolescence. Schools playa vital role in preventing students’ illegal drug use. Accordingly, we developed and evaluated a school-based, drug-use prevention program integrating the theory of planned behavior (TPB) and health literacy for junior high school students.

**Aim:**

This study aimed to use a theory-based program to prevent students from illegal drug use in Taiwanese junior high school students.

**Methods:**

We recruited 648 junior high school students aged around 13–14 years (grades 7 to 8 students) from 14 selected schools: *N* = 323 in the experimental group, *N* = 325 in the comparison group. The experimental group received 10 45-min sessions of a theory-based drug-use prevention program. The comparison group received traditional didactic teaching and drug refusal skill training. We used a generalized estimating equation (GEE) to analyze data.

**Results:**

Results of paired *t*-tests indicated that drug-use health literacy and TPB-related variables improved in the experimental group. The GEE analyses indicated that participants in the experimental group also demonstrated significantly improved health literacy (*p* < 0.001) compared to the comparison group, especially for functional (*p* < 0.001) and critical health literacy (*p* = 0.017). The experimental group also showed significant post-intervention improvement in terms of subjective norm scores (*p* = 0.024).

**Conclusion:**

Study results demonstrated the effectiveness of a drug-use prevention program on health literacy and subjective norm through integrating the Theory of Planned Behavior and health literacy. The study supports that the future implementation of similar programs for junior high school students can integrate health literacy and subjective norms as two critical program components.

**Supplementary Information:**

The online version contains supplementary material available at 10.1186/s12889-021-11830-5.

## Background

Drug use has been a critical health problem among students over the past decade. Prevalence rates for lifetime, past-year, and past-month illegal drug use were 2.79, 1.91, and 1.72%, respectively, for 15,754 senior and vocational high school students in Taiwan [1]. In Taiwan, the average age of 12–17 years old who used illegal drugs for the first time was 12.5 years old. The main reasons were curiosity, boredom and catching up with fashion [2]. A national campus survey in 2017 showed drug-use prevalence at 0.23% for junior high school students and 0.73% for senior high school students [3]. From 1999 to 2006, the prevalence of drug abuse among junior high school students was approximately between 0.6 and 1.5% [4, 5]. However, prevalence rates were relatively higher for night class students in vocational high schools. A randomized sample was drawn from 33 vocational high school night classes, which included 1079 students already employed outside the campus. Among them, 881 (81.7%) were non-drug users, 147 (13.6%) were experimental users, and 51 (4.7%) were regular users [6]. Initiating substance use at an early age is a significant predictor of later substance abuse, delinquency, and serious adverse health consequences [7, 8]. According to a national survey report in Taiwan, the first-time drug use reason was “curiosity” (70.5%), and the first-time drug use location was most often a classmate’s or friend’s home (29.9%). The survey report also indicated that the use of new types of drugs such as poisoned coffee bags, milk tea bags, and rainbow cigarettes is found mainly in young populations, and there is no gender difference, which is an emerging problem. The survey revealed that the young populations are the most common users of new types of illegal drug. Moreover, approximately90% of participants agreed that anti-drug education should be “integrated into the formal school curriculum” [9].

The theory of planned behavior (TPB) is a common theoretical framework for predicting behavior. It proposes that behavior is directly influenced by behavioral intention and perceived behavioral control (PBC), while attitude, subjective norm, and PBC can jointly influence the behavioral intention, and then indirectly influence behavior performance or maintenance [10]. In the constructs of TPB, attitude refers to the comprehensive evaluation of the target behavior, subjective norm refers to the behavior that significant others would like an individual to perform, and PBC refers to perceived difficulty and self-control in target behavior implementation. TPB was often used as a theoretical framework for illegal drug use in past studies [11]. TPB has shown satisfactory predictive power for behavior and behavioral intention [12], and TPB is suitable as the framework for illegal drug use prevention programs [13].

TPB could provide a framework for understanding students’ drug-use behavior. However, it lacks specificity on how to make behavioral changes in the context of illegal drug use [14]. Life skills can act as critical tools to assist students in rejecting drugs. These skills enable students to translate knowledge and attitude into behavior, thereby improving self-confidence, self-efficacy, positive attitude, and behavior control. They also enhance students’ ability to handle social influence [15]. A longitudinal study [16] found that life skills training was effective in reducing illegal drug use. A meta-analysis on a school-based drug-use prevention program indicated that life skills were a critical component of school-based adolescent drug prevention programs [17].

Health Literacy is defined in the Institute of Medicine report, as “the degree to which individuals have the capacity to obtain, process, and understand basic health information and services needed to make appropriate health decisions” [18]. Health literacy has received much attention in recent years. It is an important indicator of whether individuals can perform their health behaviors by obtaining, processing, and understanding basic health information and services [19]. The present study suggests that health literacy includes three components: functional, communicative/interactive, and critical literacy [20]. According to Nutbeam, functional literacy refers to skills in reading and writing that enable students to function effectively in everyday situations. Interactive literacy refers to more advanced cognitive, social, and literacy skills that can be actively used to participate in everyday activities. It is also used to extract information and derive meaning from different communication forms, and apply new information to a changing environment [21]. Critical literacy refers to more advanced cognitive skills, which are used together with social skills to critically analyze information and acquire more control over life events and situations. A systematic review investigated the relationship between health literacy and health behaviors in adolescents. The results indicated that there is a meaningful relationship between health literacy and adolescents’ substance use behaviors [19]. It implies that high health literacy may contribute to reduce substance use in adolescents.

The present study aimed to develop a drug-use prevention program incorporating health literacy to evaluate its effectiveness among junior high school students in Taiwan. We considered illegal drug-use intention as a proxy and direct variable to illegal drug-use behavior. Based on TPB, the drug-use intention could be strengthened by advancing students’ attitudes, subjective norms, and perceived control. We hypothesized that their improved health literacy would contribute to a higher level of behavioral intention to remain drug-free.

## Methods

### Participants

This study was performed in accordance with the guidelines and regulations on the Research Ethics Committee of National Taiwan University Behavior and Social Science. Aquasi-experimental design was used to recruit participants. We invited 14 junior high schools to participate in the study through the local Department of Education in seven counties and cities. We recruited two schools in each county/city and randomly assigned them to experimental and comparison groups. We invited the health education teachers of the experimental schools to attend an orientation meeting and introduced the purpose and methods of the study. After obtaining the health education teacher’s permission to participate, we invited students aged around 13–14 years (grades 7 to 8 students) from two classes of these schools to enroll in the study. Students and their parents/guardians provided written consent forms. An identical procedure is carried out for the comparison school group. Students or parents who did not provide written consent were not included in the study. All students and parents/guardians were informed of their right to participate and were assured that students’ health education grades were not contingent on participation. The final sample comprised323 and 325 students in experimental and comparison groups, respectively.

### Procedure and program delivery

**Fig. 1:**
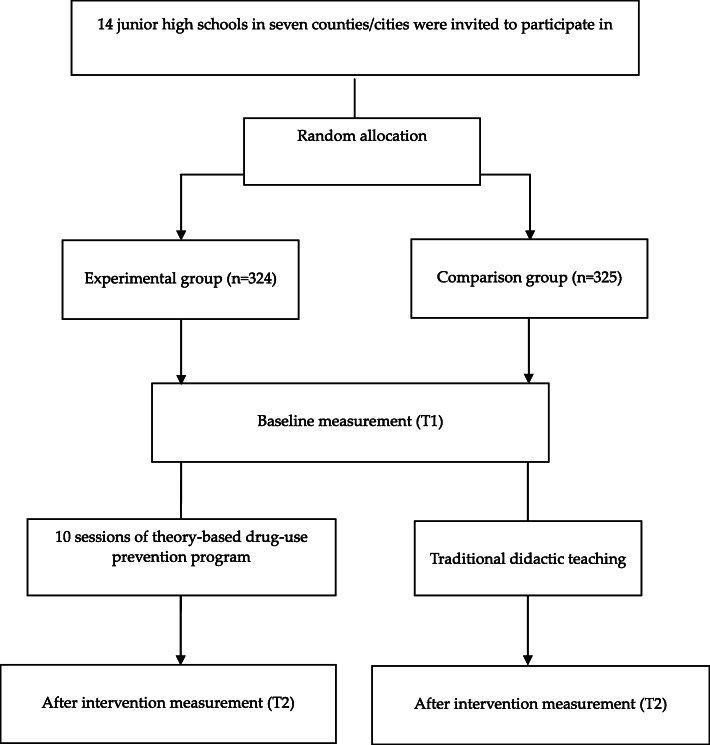
Flowchart of Participants Enrollment and Assesment

A flowchart outlining participant enrollment and assessments are presented in Fig. 1. After selecting the seven experimental schools, the research team approached the principal and health education teacher of each school to explain the research purpose, method, and protocol. After obtaining permission to conduct the study, we delivered recruitment messages to invite students to participate in this study, and scheduled an orientation meeting to ensure that the health education teachers could fully understand the purpose of the study and the cooperation works. Subsequently, we provided a half-day workshop to introduce the drug-use prevention program. In this study, we used the TPB as a framework to understand how and why students behave as they do. Further, we emphasized the learning activities to enhance the TPB variables (e.g., provision of multiple digital educational materials, learning from life scenarios and skill practice) instead of learning content. This intervention program was designed to cultivate students’ attitudes, skills, and abilities rather than only knowledge learning (Table 1). Regarding the content relationship between health literacy and the structures of planning behavior theory, we believe that TPB as a theoretical guide for understanding how and why students behave as they do. In addition to understand behavior determinants, health literacy enable students to translate knowledge into actions.

**Table 1:** Learning objectives, digital educational materials, and outcomes variables

Session	Learning objectives	Digital educational materials^**a**^	Outcome variables
1	• Understand substance use and illegal drugs • Critical thinking skills	1.Critical thinking-do not harm	• Health literacy-critical literacy • Life skills –critical thinking
2	• Recognize the various effects of illegal drugs • Recognize common new types of drugs	2.Search illegal drug information	• Anti-drug attitude • Health literacy-functional literacy
3	• Recognize common causes of illegal drug use • Decision-making skills	3.The temptation of beauty	• Health literacy-critical literacy • Life skills-decision making
4	• Recognize risk factors andspecific methods to reduce risk factors • Identify illegal drug users	4.Looking back on the initiation of drug use. E-game 1: The human puzzle	• Perceived behavioral control • Anti-drug attitude
5	• Identify high-risk situations • Effective rejection techniques	5.Refusalskills-Stand up for your position. E-game 2: Be careful! E-game 3: Refusal skill practices	• Perceived behavioral control • Life skills-refusal skills • Health literacy-interactive literacy
6	• Liability for illegal drug use • Cultivate the spirit of the rule of law	6.Stop today	• Perceived behavioral control • Anti-drug attitude
7	• Recognize protective factors and specific methods to increase protective factors	7.Classmates can help!	• Perceived behavioral control
8	• Self-stress review • Relief skills	8.Stressful pot. E-game 4: A memory test	• Perceived behavioral control • Life skills-coping with stress
9	• Know the resources for assistance and abstinence • Learning advocacy skills	9.What we want to say in those years. E-game 5: Our new anti-drug proposition	• Subjective norms • Lifeskills-negotiation skills
10	• General review and strengthening of life skills • Summary of required drug-use knowledge and skills	10.Drug Q&A Challenge	• Life skills • Perceived behavior control

^a^ Each session has a PPT related to outcome variables

The program was developed by a professional team including professionals in health promotion and health education, drug-use prevention professionals, nursing, and social workers. Teachers were strongly advised to use numerous interactive teaching methods including questions and answers, brain-storming, story-telling, case discussion, situational role-playing, game playing, watching an animated film, and to follow the discussion, value clarification, modeling, and skills practice exercises during the program implementation. To promote health literacy acquisition, we also provided program worksheets and a parent-child workbook to increase the potential practices of health literacy in daily life. Animated films, E-games, case stories, worksheets, and role-playing were used to increase health literacy learning experiences. The comparison group received the health education from the regular course one session/per week. The health education courses may or may not include illegal drug education based on the teachers’ teaching plan. In addition, Taiwan’s education policy required schools to promote anti-drug education activities per semester, usually through lecture forms, so student might receive at least one public speech every semester. Anti-drug lectures usually provide students with the latest knowledge about illegal drugs, strengthen their anti-drug attitudes, and advise students not to use illegal drugs.

**Fig. 2:**
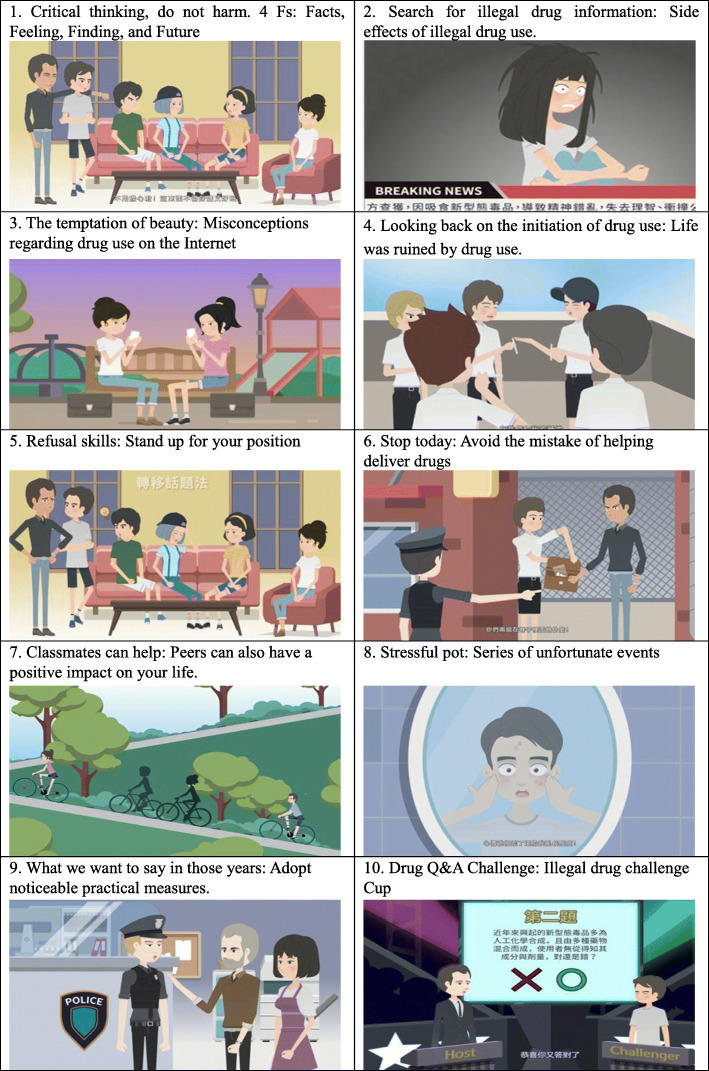
Digital educational materials: 10 animated E-games

**Fig. 3:**
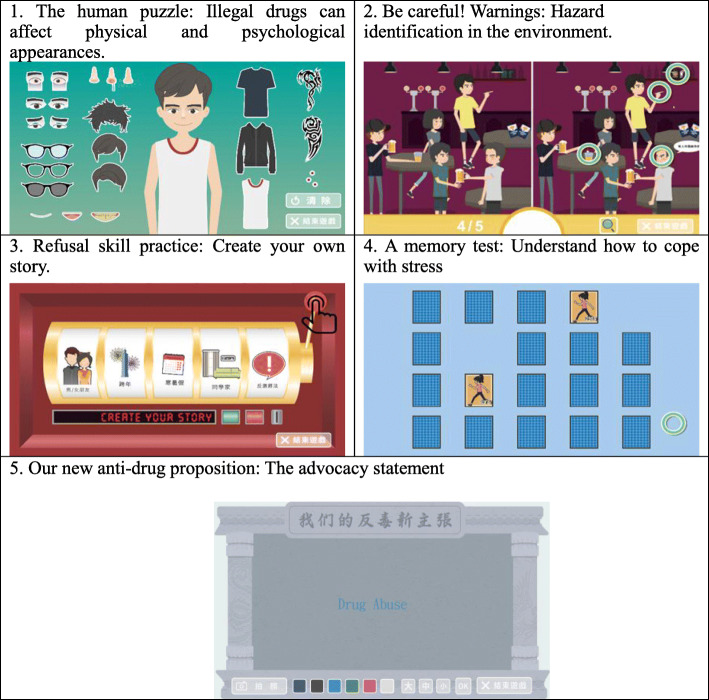
Digital educational materials: 5 E-games

Before program implementation, the relevant materials and worksheets were implemented in a junior high school as a pilot test to ensure their appropriateness. The program consisted of 10 45-min sessions. The sessions were delivered in a health education class, morning study time, and during the class meeting time, which was arranged by the health education teacher and school administration. The 10 sessions were completed within 6 months. A structured self-reported questionnaire was administered to students at baseline and the end of the program by research staff blind to the students’ group status. The principal investigator supervised the teachers during program implementation to ensure fidelity to program design. A regular monthly meeting was scheduled to support health education teachers’ program delivery (Figs. 2 and 3).

### Instruments

The four TPB variables including attitude, subjective norm, PBC, and intention to not use illegal drugs were modified from the previous study with permission [16]. Demographic variables consisted of gender, parents’/guardians’ education level, parenting style (authoritative vs. democratic), household status (living with both parents, living with a single parent, and others), and lifetime substance use (smoking, drinking, and betel nuts chewing).

Drug-use-related health literacy measures students’ ability to access and understand information and resources of substance use prevention and apply them to make the right decisions to maintain and promote their health [21]. There are 14 questions in total, and the overall Cronbach’s α = 0.86; the functional literacy are 5 questions, interactive literacy are 2 questions, and critical literacy are 7 questions. This scale consists of five-point Likert-type items, ranging from 1 (*strongly disagree*) to 5 (*strongly agree*). Items appear as five scenario based on the logic flow of drug-use paragraphs, respectively.

A sample scenario is “When the school had activities, Leo heard the discipline directorsay to all students: In recent years, Taiwan’s illegal drug-use has escalated according to the news, and the average age of users has gradually decreased. Students should pay attention not to go to at-risk environments such as Internet cafes, billiards rooms, and home parties, to prevent exposure to illegal drugs in the community. If you have family and friends with a drug use problem who need help, please dial 0800-775-885.” The three follow-up items after that paragraph are “If I were the main character, I would reduce my access to at-risk places;” “I know what kinds of places are ‘at-risk environments’ that may expose me to illegal drugs;” and “I know that the ‘special line for successful detoxification’ is 0800-770-885, and I will support my family and friends in need of those resources.”The higher the score, the higher level of drug use-related literacy.

Attitude was measured using four pairs of evaluative bipolar adjectives (pairs of opposite terms) to assess students’ positive or negative evaluations and feelings regarding illegal drug use. Each item was scored on a Likert-type scale with a reversed score scale of 1–5, with higher scores indicating a higher level of agreement on not using drugs. A sample item is “To me, drug use makes me feel joyless/joyful.” Exploratory factor analysis extracted only one factor and the factor could explain 75.47% of the variance. The Cronbach’s α coefficient was 0.80 in this study.

Subjective norm was measured by five items using a five-point Likert-type scale. Each item was scored from 1 to 5 with higher scores indicating a higher level of significant others’ agreement on not using drugs. A sample item was “My teachers don’t think I should use drugs.” Exploratory factor analysis extracted only one factor and the factor could explain 66.83% of the variance. The Cronbach’s α coefficient was 0.93 in this study.

Perceived behavior control was measure dusing two items rated on a five-point Likert-type scale. Each item was scored from 1 to 5 with higher, scores indicating a higher level of students’ confidence in not using drugs. A sample item is “I am confident I won’t use drugs.” Exploratory factor analysis extracted only one factor and the factor could explain 85.88% of the variance. Cronbach’s α coefficient was 0.83 in this study.

We used the intention not to use drugs as a proxy measure for drug-free behavior because most students had not used any drugs before. The intention not to use drugs was measured using three items rated on a five-point Likert-type scale. Each item was scored from 1 to 5 with higher scores indicating a higher level of students’ agreement on not using drugs. A sample item is “I would not like to use drugs.” Exploratory factor analysis extracted only one factor, which could explain 86.48% of the variance. Cronbach’s α coefficient was 0.93 in this study.

Please see the Additional file 1 for detail information about study measures.

### Statistical analysis

SPSS version 22.0 was used for the Descriptive analyses of the demographic and outcome variables. Chi-square tests were used to compare percentages on the demographic status between the experimental and comparison groups. Under the context of comparison group also received a certain degree of educational intervention, we used paired-t tests to examine the pure program effects for experimental group without considering the comparison group. The group comparisons of outcome measures at baseline were determined by performing Hotelling’s T2 to avoid inflating type I error. We applied the pair-t test to exam the improvements of outcome variables within group. Then, we conducted further analysis of generalized estimating equation (GEE) to explore the intervention effects. GEE was used to investigate the effects of time, groups, and their interactions on the outcome variables. GEEs enabled understanding the patterns of the time change and the effects at both the individual and group levels. Moreover, we used GEE to examine the relative program effects of experimental versus comparison groups because when promoting this program in the future, we will still face the problem of students receiving a certain degree of educational intervention. GEEs enabled understanding the patterns of the time change and the effects at both the individual and group levels.

## Results

There were no statistically significant differences between groups in terms of participants’ gender, parents’/guardians’ education level, parenting style (authoritative, democratic, and others), household status (living with both parents, living with a single parent and others), and substance lifetime use (smoking, drinking, and betel nuts chewing) (Table 2).

**Table 2:** Participants of experimental and comparison groups at baseline

	Experimental		Group Comparison			
	(*N* = 323)		Group			
			(***N*** = 325)		χ2	***p***
	*N*	%	*N*	%		
Gender^a^					0.97	0.325
Male	169	52.48	158	48.62		
Female	153	47.52	167	51.38		
Parents’/Guardians’ Education Level ^a^					1.16	0.560
9 Years	60	19.42	61	19.55		
12 Years	115	37.22	128	41.03		
College & Graduate School	134	43.36	123	39.42		
Parenting Style ^a^					3.66	0.160
Authoritative	34	10.63	50	15.63		
Democratic	277	86.56	263	82.19		
Others	9	2.81	7	2.18		
Household status					0.76	0.684
Living with both parents	244	75.55	245	75.38		
Living with a single parent	63	19.50	68	20.92		
Others	16	4.95	12	3.70		
Substance lifetime use					2.760	0.097
Yes	79	24.46	62	19.08		
No	244	75.54	263	80.92		

^a^ Some participants in the experimental and comparison group did not answer this question

### Improvements of outcome variables

Results of paired *t*-tests indicated that drug-use health literacy and TPB-related variables improved after intervention for the experimental group. The paired *t*-tests and *p*-values of drug-use health literacy, attitude, subjective norm, perceived behavior control and behavioral intention were 7.03 (*p* < 0.001), 2.43 (*p* = 0.015), 2.16 (*p* = 0.032), 2.46 (*p* = 0.014), and 2.85 (*p* = 0.005), respectively. Although the mean differences in health literacy and TPB-related variables between pre- and post-intervention slightly improved for the comparison group except for subjective norm (23.35 vs. 23.19),the results of the paired *t*-tests for the comparison group were all not significant.

Group differences in patterns of change over time are shown in Table 3. Results of GEE analyses indicated that the experimental group made significant improvements compared to the comparison group in health literacy and subjective norm scores but not for attitude, perceived behavior control, or behavioral intention. There was a significant group x time interaction for health literacy and subjective norm. The experimental group showed an improvement in health literacy score (coefficient = 2.01, Wald χ^2^ = 20.39, *p* < 0.001) and subjective norm (coefficient = 0.61, Wald χ^2^ = 5.07, *p* = 0.024).

**Table 3:** Results of GEE ^a^ analyses for outcome variables

	Coefficient	SE	Wald χ2	*p*
Drug-use related health literacy (all)				
Group (Experimental group) ^b^	−1.25	0.48	6.78	0.009
Time (Post-test) ^c^	0.35	0.31	1.29	0.257
Group (Experimental) x Time (Post-test) ^d^	2.01	0.45	20.39	**< 0.001**
Drug use related functional literacy				
Group (Experimental) ^b^	−0.69	0.22	9.84	0.002
Time (Post-test) ^c^	0.30	0.14	4.50	0.034
Group (Experimental) x Time (Post-test) ^d^	1.15	0.22	27.39	**< 0.001**
Drug use related communicative/interactive literacy				
Group (Experimental) ^b^	−0.02	0.08	0.05	0.821
Time (Post-test) ^c^	0.06	0.06	0.86	0.353
Group (Experimental) x Time (Post-test) ^d^	0.04	0.08	0.27	0.602
Drug use related critical literacy				
Group (Experimental) ^b^	−0.55	0.27	4.23	0.040
Time (Post-test) ^c^	0.13	0.18	0.49	0.485
Group (Experimental) x Time (Post-test) ^d^	0.63	0.27	5.72	**0.017**
Attitude				
Group (Experimental) ^b^	0.35	0.21	2.63	0.105
Time (Post-test) ^c^	0.29	0.19	2.29	0.130
Group (Experimental) x Time (Post-test) ^d^	0.08	0.24	0.12	0.727
Subjective norm				
Group (Experimental) ^b^	−0.90	0.24	14.52	< 0.001
Time (Post-test) ^c^	−0.12	0.16	0.61	0.433
Group (Experimental) x Time (Post-test) ^d^	0.61	0.27	5.07	**0.024**
Perceived behavior control				
Group (Experimental) ^b^	−0.28	0.10	7.91	0.005
Time (Post-test) ^c^	0.04	0.07	0.31	0.580
Group (Experimental) x Time (Post-test) ^d^	0.15	0.10	2.12	**0.145**
Behavioral intention				
Group (Experimental) ^b^	−0.15	0.14	1.22	0.269
Time (Post-test) ^c^	0.14	0.09	2.25	0.134
Group (Experimental) x Time (Post-test) ^d^	0.17	0.14	1.42	**0.233**

^a^ GEE: generalized estimating equation

^b^ Reference group (group): comparison group

^c^ Reference group (time): pretest

^d^ Reference group (group x time): comparison group pretest

Group differences in patterns of change over time in Table 3 also indicated that the experimental group made significant improvements compared to the comparison group in term of the scores on functional (coefficient = 1.15, Wald χ^2^ = 27.39, *p* < 0.001) and critical literacy (coefficient = 0.63, Wald χ^2^ = 5.72, *p* = 0.017) but not for communication/interactive literacy (coefficient = 0.04, Wald χ^2^ = 0.27, *p* = 0.602).

## Discussion

Results of paired t-tests indicated that drug-use health literacy and TPB-related variables improved in the experimental group. The GEE analyses indicated that participants in the experimental group also demonstrated significantly improved health literacy compared to the comparison group, especially for functional and critical health literacy. Our findings supported the effectiveness of a school-based, drug-use prevention program integrating health literacy developed for and evaluated by junior high school students in Taiwan. Compared with aprevious study [16], our study has better external generalizability because participants were recruited from seven counties/cities. The sampling method will enable researchers to make inferences about this population. Our findings provided evidence for combining a psychosocial construct and health literacy to prevent student drug use. A previous systematic review on health literacy in childhood and youth indicated health literacy in children and young people is described as comprising variable sets of key dimensions, each appearing as a cluster of related abilities, skills, commitments, and knowledge that enable an individual to approach health information competently and effectively and to make health-promoting decisions [22]. Our program content and teaching methods provided participants in the experimental group a cluster of drug-use-related knowledge, skills, beliefs, commitmentnorms not to use illegal drugs through interactive teaching methods. To the best of our knowledge, this is the first study to increase students’ drug-use-related health literacy in Taiwan. The program was unique in its integration of the TPB and health literacy.

The participants in the experimental group made significant improvements compared to their counterparts of the comparison group after an intervention on functional and critical literacy, but not in communication/interactive literacy. Health literacy is an ability of how to make behavioral changes. We expected the integration of TPB and health literacy increasing students’ ability to refuse illegal drugs. However, our finding shows that the cultivation of communicative/interactive literacy takes time, and the duration of this program was limited. Therefore, it is not surprising that the communicative/interactive literacy improved, but it was not statistically significant. The effectiveness of length of intervention or different learning activities should be further explored in the future.

As long as the program duration is extended and the time devoted to peer interaction increased, the intervention effectiveness is promising. Drug-use-related health literacy is critical because it enabled students to acquire and understand information and resources regarding drug use prevention. Students can use this information and resources to make personal decisions to maintain and promote their drug-free status. Currently, the health literacy scales mainly focused on medical-related topics [23]. This program provided a new research direction to support the integration of health literacy into the drug-use prevention program and promote adolescent health. Recently, adolescents are more likely to be exposed to illegal drugs through internet access or social media. Adolescents may receive persuasive messages to convince them to initiate drug use when they interact with others on the Internet [24]. At this time, it is critical to have enough health literacy to teach students how to verify and assess a large amount of information available on the Internet or social media. It is also important to teach adolescents how to correctly and precisely discuss and communicate information about drug use, and make drug-free decisions, to reduce illegal drug use. Communication/interactive health literacy needs to be emphasized in future drug use prevention programs, and it needs a longer duration to avoid one-way indoctrination.

In addition to health literacy, this study also evaluated the effectiveness of students’ attitudes, subjective norms, perceived behavior control, and behavioral intention. The results showed that the changes in attitude, subjective norms, perceived behavior control, and behavioral intention in the experimental group made significant improvements, which was in line with a previous study [25], especially the subjective norms, which showed significant improvement when compared to the comparison group. It suggested the study can successfully assist students to perceive significant others’ norms that disapprove of illegal drug use. It is critical for junior high school students because most drug users initiate drug use during adolescence. A previous study explored the “Drugs-at-work” program designed for students’ normative beliefs and found that it could effectively reduce students’ intention to use drugs [26]. A positive normative belief in students can contribute to a positive climate of drug use rejection on campus.

There were also improvements in experimental students’ attitude, perceived behavior control, and behavioral intention, but there was no significant difference when compared with the comparison group. The first possible reason was that students’ baseline scores were surprisingly high, which led to limited room for improvement in the post-test, similar to a ceiling effect (a ceiling effect is said to occur when a high proportion of participants) in a study have maximum scores on the observed variable [27]. The second possible reason was that there was only one health education session per week for students in grades 1 to 9 in Taiwan, and drug use content was included as an essential component in the health education curriculum. The component emphasized increasing students’ anti-drug knowledge, attitude, and refusal skills. Participants in the comparison group also benefited from learning in a regular health education class and revealed slight improvements on outcome variables. Therefore, students’ anti-drug knowledge, attitudes and behavioral intentions may be improved. Furthermore, some psychosocial variables, such as attitude, perceived behavior control, and behavior intention might requirea long time to be improved. The previous study also revealed that the prevalence of illicit drug use among adolescents tended to increase with age [6, 28], which indicated the necessity of continuing to promote drug-use prevention programs in junior high schools.

Integrating health literacy into a theory-based drug-use prevention program is effective. Program components delivered by digital animated files and E-games can increase students’ interest in learning, strengthen interactions between teachers and students during the teaching process, and reduce the burden on teacher’s preparation time. The study provides an evidence-based drug-use prevention program for junior high school students with a focus on health literacy. It is believed that increasing the students’ anti-drug health literacy will benefit their future life and have a higher probability of reducing drug use.

## Conclusion

Study results demonstrated the effectiveness of a drug-use prevention program on health literacy and subjective norm by integrating the Theory of Planned Behavior and health literacy. The study supports that the future implementation of similar programs for junior high school students can integrate health literacy as a critical program component.

## Supplementary Information

**Additional file 1.**

## Data Availability

The datasets used and/or analyzed during the current study are available from the corresponding author on reasonable request.

## Abbreviations

TPB: Theory of planned behavior
GEE: Generalized estimating equation
PBC: Perceived behavioral control

## References

[1] Liao JY, Huang CM, Lee CTC, Hsu HP, Chang CC, Chuang CJ, Guo JL (2018). Risk and protective factors for adolescents’ illicit drug use: a population-based study. Health Educ J.

[2] Ministry of Health and Welfare (MOHW). National health interview and drug abuse survey. 2017. https://www.hpa.gov.tw/Pages/Detail.aspx?nodeid=363&pid=6534.

[3] Guo JL, Huang CM, Lee TC (2017). National web-based survey of illegal drug use among adolescents.

[4] Chou P, Liou M, Lai M, Hsiao M, Chang H (1999). Time trend of substance use among adolescent students in Taiwan, 1991-1996. J Formosan Med Assoc.

[5] Chen WJ, Fu TC, Ting TT, Huang WL, Tang GM, Hsiao CK, Chen CY (2009). Use of ecstasy and other psychoactive substances among school-attending adolescents in Taiwan: national surveys 2004–2006. BMC Public Health.

[6] Huang CM, Lin LF, Lee TC, Guo JL (2013). Proximal to distal correlates of the patterns of illicit drug use among night school students in Taiwan. Addict Behav.

[7] Trujillo CA, Obando D, Trujillo A (2019). An examination of the association between early initiation of substance use and interrelated multilevel risk and protective factors among adolescents. PLoS One.

[8] Gil AG, Wagner EF, Tubman JG (2004). Associations between early-adolescent substance use and subsequent young-adult substance use disorders and psychiatric disorders among a multiethnic male sample in South Florida. Am J Public Health.

[9] Food and Drug Administration, MOHW (2018). National survey report of illegal drug use in Taiwan.

[10] Ajzen I, Kuhl J, Beckmann J (1985). From intentions to actions: atheory of planned behavior. Action Control: From Cognition to Behavior.

[11] Ram SS, Hussainy S, Henning M, Stewart K, Jensen M, Russell B (2017). Attitudes toward cognitive enhancer use among New Zealand tertiary students. Subst Use Misuse.

[12] Armitage CJ, Conner M (2001). Efficacy of the theory of planned behaviour: a meta-analytic review. Br J Soc Psychol.

[13] Huang CM, Chien LY, Cheng CF, Guo JL (2012). Integrating life skills into a theory-based drug-use prevention program: effectiveness among junior high students in Taiwan. J Sch Health.

[14] Sharma M, Kanekar A (2007). Theory of reasoned action & theory of planned behavior in alcohol and drug education. J Alcohol Drug Educ.

[15] Bühler A, Schröder E, Silbereisen RK (2007). The role of life skills promotion in substance abuse prevention: a mediation analysis. Health Educ Res.

[16] Guo JL, Lee TC, Liao JY, Huang CM (2015). Prevention of illicit drug use through a school-based program: results of a longitudinal, cluster-randomized controlled trial. J Adolesc Health.

[17] Tobler NS, Roona MR, Ochshorn P, Marshall DG, Streke AV, Stackpole KM (2000). School-based adolescent drug prevention programs: 1998 meta-analysis. J Prim Prev.

[18] National Library Medicine (2019). Health Literacy.

[19] Fleary SA, Joseph P, Pappagianopoulos JE (2018). Adolescent health literacy and health behaviors: a systematic review. J Adolesc.

[20] Nutbeam D (2000). Health literacy as a public health goal: a challenge for contemporary health education and communication strategies into the 21st century. Health Promot Int.

[21] Nutbeam D (2008). The evolving concept of health literacy. Soc Sci Med.

[22] Bröder J, Okan O, Bauer U, Bruland D, Schlupp S, Bollweg TM, Saboga-Nunes L, Bond E, Sørensen K, Bitzer EM, Jordan S, Domanska O, Firnges C, Carvalho GS, Bittlingmayer UH, Levin-Zamir D, Pelikan J, Sahrai D, Lenz A, Wahl P, Thomas M, Kessl F, Pinheiro P (2017). Health literacy in childhood and youth: a systematic review of definitions and models. BMC Public Health.

[23] Mackert M, Mabry-Flynn A, Champlin S, Donovan EE, Pounders K (2016). Health literacy and health information technology adoption: the potential for a new digital divide. J Med Internet Res.

[24] Gobbi G, Atkin T, Zytynski T, Wang S, Askari S, Boruff J, Ware M, Marmorstein N, Cipriani A, Dendukuri N, Mayo N (2019). Association of cannabisuse in adolescence and risk of depression, anxiety, and suicidality in young adulthood: asystematic review and meta-analysis. JAMA Psychiatry.

[25] Eisen M, Zellman GL, Murray DM (2003). Evaluating the lions–quest “skills for adolescence” drug education program: second-year behavior outcomes. Addict Behav.

[26] Wright LS (2007). A norm changing approach to drug prevention. J Drug Educ.

[27] van der Putten JJMF, Hobart JC, Freeman JA, Thompson AJ (1999). Measuring change in disability after inpatient rehabilitation: comparison of the responsiveness of the barthel index and the functional independence measure. J Neurol Neurosurg Psychiatry.

[28] Chang CC, Liao JY, Huang CM, Hsu HP, Chen CC, Guo JL (2018). Evaluation of the effects of a designated program on illegal drug cessation among adolescents who experiment with drugs. Substance Abuse Treat Prev Policy.

