# Supplementary material for: Integrating health literacy into a theory-based drug-use prevention program: a quasi-experimental study among junior high students in Taiwan
Source: BMC Public Health. 2021 Sep 28;21:1768. doi: 10.1186/s12889-021-11830-5 (PMC8479915; doi:10.1186/s12889-021-11830-5)
Supplement: Supplementary file 1 — Additional file 1. [file 12889_2021_11830_MOESM1_ESM.docx]

Appendix. The study measures

|  | | Likert-type items, ranging from 1 (strongly disagree) to 5 (strongly agree) |
| --- | --- | --- |
| 1.Drug-use related health literacy | |  |
|  | functional literacy(n=5) | - If I were the main character, I would reduce my access to at-risk places. - I know what kinds of places are ‘at-risk environments’ that may expose me to illegal drugs. - I know that the ‘special line for successful detoxification’ is 0800-770-885, and I will support my family and friends in need of those resources. - For the "carnival party" mentioned in the advertisement, I know where to search for information to check if it is safe. - If I were the main character, I know how to find helpful resources (for example, teachers or classmates) when I am too tired or upset. |
|  | interactive literacy(n=2) | - If I were the main character, when the senior said, "I am taking these pills myself, there is no problem!", I have learned relevant skills to refuse the senior's invitation. - If I were the main character, I know how to explain his friend’s problem to the teacher, and assist and support his friend to stay away from illegal drugs. |
|  | critical literacy(n=7) | - I think "mysterious energy keeps you HIGH" is a trick to induce the use of illegal drugs. - "Participating in a carnival party will make the mood very high" and "a rare opportunity" are all advertising techniques, and only the good side is said. - I know that there are many ways to satisfy my pursuit of an adventurous and exciting life, instead of choosing to participate in this "carnival party". - When others offer me pills of unknown ingredients, I will be more vigilant. - If I were the main character, and discovered that the senior student "mysteriously took an unknown pill...he was full of energy after taking it...a strange smile appeared", I would be able to judge the senior student may have used illegal drugs. - If I were the main character, I found a friend "has changed from overwhelmed to high, and mysteriously went to the Internet cafe to find a certain person who is suspected to buy drugs..." I could have the ability to judge this person may have used illegal drugs. - If I were the main character, I knew how I might react when I was stressed, and I knew how to relieve it. |
| 2.Attitude(n=4) | | - Use illegal drugs is unpleasant for me. - Use illegal drugs is bored for me. - Use illegal drugs is useless for me. - Use illegal drugs is not good for me. |
| 3.Subjective norm(n=5) | | - I feel very stressed when someone invites me to take illegal drugs. - My parents or relatives think that I should not use illegal drugs. - My teachers think that I should not use illegal drugs. - Classmates in the school (all the students in all grades are included) think that I shouldn't use illegal drugs. - Friends outside school think that I shouldn’t use illegal drugs. |
| 4. Perceived behavior control(n=2) | | - I am confident that I will not use illegal drugs. - It is easy for me not to use illegal drugs. |
| 5.Behavioral intention(n=3) | | - I don't want to use illegal drugs now. - I don't want to use illegal drugs in the future. - I will not use the illegal drug if I have the opportunity to come into contact with the illegal drug in the future. |
